# Nationwide improvement in outcomes of emergency admission for ulcerative colitis in England, 2005‐2013

**DOI:** 10.1111/apt.15315

**Published:** 2019-05-28

**Authors:** Mustafa Shawihdi, Susanna Dodd, Constantinos Kallis, Pete Dixon, Ruth Grainger, Stuart Bloom, Fraser Cummings, Mike Pearson, Keith Bodger

**Affiliations:** ^1^ Department of Biostatistics Institute of Translational Medicine, University of Liverpool Liverpool UK; ^2^ Gastrointestinal Service University College London Hospital London UK; ^3^ Gastroenterology Unit, University Hospital Southampton NHS Trust Southampton UK; ^4^ Digestive Diseases Centre Aintree University Hospital NHS Trust Liverpool UK

## Abstract

**Background:**

The UK IBD Audit Programme reported improved inpatient care processes for ulcerative colitis (UC) between 2005 and 2013. There are no independent data describing national or institutional trends in patient outcomes over this period.

**Aim:**

To assess the association between the outcome of emergency admission for UC and year of treatment.

**Methods:**

Retrospective analysis of hospital administrative data, focused on all emergency admissions to English public hospitals with a discharge diagnosis of UC. We extracted case mix factors (age, sex, co‐morbidity, emergency bed days in last year, deprivation status), outcomes of index admission (death and first surgery), 30‐day emergency readmissions (all‐cause, and selected causes) and outcome of readmission.

**Results:**

There were 765 deaths and 3837 unplanned first operations in 44 882 emergency admissions, with 5311 emergency readmissions (with a further 171 deaths and 517 first operations). Case mix adjusted odds of death for any given year were 9% lower (OR 0.91, 95% CI: 0.89‐0.94), and that for emergency surgery 3% lower (OR 0.97, 95% CI: 0.95‐0.98) than the preceding year. Results were robust to sensitivity analysis (admissions lasting ≥4 days). There was no reduction in odds for all‐cause readmission, but rates for venous thromboembolism declined significantly. Analysis of institutional‐level metrics across 136 providers showed a stepwise reduction in outliers for mortality and unplanned surgery.

**Conclusions:**

Risk of death and unplanned surgery for UC patients admitted as emergencies declined consistently, as did unexplained variation between hospitals. Risk of readmission was unchanged (over 1 in 10). Multiple factors are likely to explain these nationwide trends.

## 1 INTRODUCTION

Mortality for people with ulcerative colitis (UC) does not differ significantly from the general population.^1^ However, severe disease exacerbations requiring hospitalisation can be life‐threatening.^2^ Reducing the risk of in‐hospital mortality requires optimal assessment, best supportive care, appropriate escalation of immunosuppressants and (where drugs fail or complications arise) timely surgical intervention.^2^, ^3^ High‐quality care should eliminate avoidable deaths and reduce the need for unplanned, life‐changing emergency surgery.

Over a 10‐year period, a nationwide quality improvement programme (the UK IBD Audit) was implemented in Britain, focusing particularly on inpatient care.^4^, ^5^, ^6^, ^7^ Explicit standards were defined and four rounds of audit were conducted (2006‐2013).^4^, ^5^, ^6^, ^7^ The inaugural round showed significant shortfalls in emergency care and wide variation in service provision.^4^, ^8^ Forty two deaths were reported among 2767 admissions for colitis—a crude mortality rate of 1.7%.^4^ Directly comparable data from other countries were limited, although expert centres were reporting in‐hospital mortality rates below 1% for acute severe colitis at that time.^9^ Analysis of 2989 admissions for UC in the USA (1994‐2006) revealed crude mortality of 1.2%,^10^ whereas death rate among 4278 patients admitted to hospitals in Ontario (2002‐2008) was just 0.75%.^11^

Participation in the UK audit grew to 95% of organisations before the programme ended.^7^ Step‐wise improvements in service organisation and care processes were observed. Alrubaiy et al summarised trends for the first three rounds (4937 emergency UC admissions),^8^ finding increasing rates of ward review by IBD specialist nurses (from 24% to 45%), stool testing for *Clostridium difficile* (54%‐75%) and heparin thromboprophylaxis (54%‐75%). By the fourth round, overall performance for these indicators was 48%, 76% and 90%, respectively.^7^ The use of salvage drug therapies for steroid‐refractory colitis rose (31%‐65%) and the contribution of anti‐TNF agents increased eightfold.^4^, ^7^, ^8^ Crude rates of unplanned surgery across the four audits were 12.8%, 12.5%, 12.1% and 12%, respectively, but the statistical significance of this trend was not reported. However, the audit did report a significant decline in crude mortality rate (1.7%, 1.5%, 0.8% and 0.75%, respectively).^4^, ^5^, ^6^, ^7^ Although methodological limitations were acknowledged, this suggested that emergency care for colitis had improved substantially in the UK, with a possible halving of in‐hospital death rate.^8^

There is growing international interest in quality improvement and benchmarking of IBD care within individual health care systems and between countries.^12^, ^13^, ^14^, ^15^ The UK IBD Audit has served as a model for a recent national programme in Australia.^16^ However, the interpretation of trends in outcomes from serial UK audits is difficult owing to differences in site participation and case ascertainment between audit rounds and different hospitals. Such limitations preclude any meaningful comparison of case mix‐adjusted outcomes across time at national level. Furthermore, small counts of deaths and emergency surgical events did not allow for analysis of whether inter‐institutional variation in patient outcomes was narrowing over time.

There are no independent, nationally representative data published to describe real‐world outcomes, time trends or institutional variation for emergency UC care over the decade when the UK IBD audit was active. To address this knowledge gap, we analysed routinely collected administrative data for England.

Our primary aim was to establish whether outcomes of emergency hospital admission for UC improved in England between 2005 and 2013, as measured by risk of in‐hospital death, emergency surgery and unplanned readmission within 30 days of discharge. Our secondary aims were to explore whether improvements were seen across all regions of England and to establish whether inter‐institutional variation and the prevalence of outliers for key metrics reduced over this period. We also examined the diagnostic profile of readmissions and changes over time for selected categories.

## 2 MATERIALS AND METHODS

### 2.1 Design and sources of data

This was a retrospective analysis of routinely collected hospital administrative data for England. We analysed Hospital Episode Statistics (HES), a collection of anonymised patient data managed by the Department of Health in England.^17^ The admitted patient care database includes information on all inpatient hospital stays for public NHS hospitals, including all institutions admitting emergency cases. Each admission (spell) consists of a number of episodes covering a period of care under different consultants (specialists). Clinical content in HES comprises a list of primary and secondary diagnoses (based on the International Classification of Diseases Tenth Version, ICD‐10) and procedures (OPCS Classification of Interventions and Procedures version 4, OPCS‐4) generated by local clinical coders from hospital records after discharge. Medication is not recorded.

This work was undertaken in partnership with the UK IBD Registry^18^ to support the development and reporting of national and institutional level benchmarking metrics for IBD care. HES data were provided by NHS Digital (formerly the Health & Social Care Information Centre) for all patients with a discharge diagnosis of IBD between 2004/05 and 2013/2014, including their all‐cause hospital admissions. Parts of the data presented here were shared with participating hospitals as local‐level HES reports.^19^ We also extracted data on crude rates of in‐hospital mortality for all‐cause emergency admissions to English hospitals for each fiscal year from a published source.^20^

### 2.2 Patient population and their all‐cause hospital admissions

The target population was adult patients (>16 years) having one or more non‐elective admissions to an English hospital with a primary discharge diagnosis of ulcerative colitis (ICD‐10 codes: K51.0, K51.1, K51.2, K51.3, K51.4, K51.5, K51.8 and K51.9). This list corresponds to codes used to identify samples of admissions for the UK IBD Audit.^4^ We refer to these admissions as UC‐specific emergency admissions. The main cohort included all patients with a completed discharge between 1 April 2005 and 31 March 2014.

We used the 2004/2005 fiscal year as a screening year to ensure that every UC‐specific admission in the analysis would have at least 12 months of retrospective data available. This allowed us to extract all hospital events in the year before each index admission. Hence, the cohort included all persons discharged with a primary diagnosis of UC across nine fiscal years (2005/06 to 2013/14). We excluded patients with any occurrence of diagnosis codes for Crohn's disease or colorectal cancer.

To track patient journeys, we extracted all hospital admissions across 9 years and ordered them chronologically for each case. Key events were flagged, including emergency admissions for any reason (referred to as all‐cause emergency admissions). For each UC‐specific emergency admission, we identified any unplanned readmission within 30‐days of discharge (all‐cause 30‐day emergency readmissions).

### 2.3 Demographic, co‐morbidity and socioeconomic variables

For each admission, we extracted data for age and sex. All secondary diagnostic fields were screened for comorbidities using ICD‐10 codes from the Charlson index, creating a categorical comorbidity variable (none, 1 or ≥2 comorbidities) as previously described.^17^ Each episode in HES contains a deprivation variable for place of residence. This allows ranking of areas from most to least deprived using the Indices of Multiple Deprivation for England, which we grouped into quintiles.^17^ A further case mix variable was generated to derive the sum of all‐cause emergency bed days in the 12 months prior to each admission, to reflect overall patient morbidity in the preceding year.

### 2.4 Definition for first major surgery for ulcerative colitis

All hospital admissions were screened for any instance of coding that would be consistent with a patient's first major surgical intervention for colitis. We generated a code list for all gastrointestinal surgical procedures recorded for the cohort, then selected all OPCS‐4 codes that were compatible with a primary operation (ie, colectomy).^21^ For each case, their first (earliest) instance of a relevant primary operation was recorded, and this was further flagged when this occurred during an emergency admission (first major emergency surgery). For each patient undergoing surgery, we calculated the number of days from admission to procedure date (time to surgery). We also identified whether additional procedure codes were recorded to indicate a laparoscopic approach.^21^

### 2.5 Outcome measures

#### 2.5.1 Events during UC‐specific admissions

Key outcomes of interest were acute events during the UC‐specific emergency admission, namely in‐hospital death and first major emergency surgery. We also defined a composite outcome, surgery‐free discharge, based on the absence of either event during the index admission. For all analyses of risk for first major surgery, only patients without prior surgery were included—ie, only patients remaining at risk for a colectomy were included.

#### 2.5.2 All‐cause 30‐day emergency readmissions

We also examined unplanned care in the immediate post‐discharge period. This focussed on re‐admission for any reason within 30 days of discharge (all‐cause 30‐day emergency readmission), and the occurrence of major outcomes during those readmissions (in‐hospital death and first major surgery). We aggregated in‐hospital deaths and surgical events across both index admissions and 30‐day readmissions to create composite outcome variables.

#### 2.5.3 Cause‐specific 30‐day emergency readmissions

Reasons for readmission were examined by analysing primary diagnosis codes. Readmissions were stratified according to whether surgery had occurred during index admission, aggregated by ICD‐10 code and ranked by frequency. To examine time trends in specific reasons for readmission, we selected two categories based on their frequency and relevance to the UK IBD Audit. First, we flagged emergency readmissions for venous thromboembolism (VTE) which was relevant to the audit's focus on driving increased use of heparin prophylaxis.^4^, ^5^, ^6^, ^7^ Secondly, we identified readmissions for any major infection. Increasing use of salvage therapies may have increased the risk of early readmission for infections. To classify readmissions, we used a published classification system which identifies “baskets” of primary diagnoses.^22^ This includes code lists for VTE, and a series of categories for specific infections which we pooled together into one group (sepsis, pneumonia and upper respiratory infections, urinary tract, skin/soft tissue and bone, meningitis and unspecified bacterial infections).

### 2.6 Statistical analysis and models

#### 2.6.1 Descriptive statistics

We aggregated data for each fiscal year, generating counts of admissions and patients, summarising case mix and calculating crude rates for each outcome. For selected analyses, the 9‐year observation period was divided into three 3‐year periods (fiscal years 2005/2006 to 2007/2008, 2008/2009 to 2010/2011 and 2011/2012 to 2013/2014) with aggregation of admissions and outcomes for each period, with trends in categorical variables tested by chi square and continuous variables by ANOVA as appropriate. Case mix adjusted time trends in outcomes at national level were explored in multivariable models, as described below.

#### 2.6.2 Multivariable modelling of national time trends in outcomes

A series of multivariable logistic models explored the primary question as to whether case‐mix adjusted outcomes were significantly improving over the course of the observation period at national level. Hence, the exposure variable of interest was fiscal year of admission, with a model for each of the outcomes (dependent variables). Case mix variables included age, sex, co‐morbidity, deprivation status, the number of all‐cause emergency bed days in the preceding year and (where relevant) the occurrence of first major surgery during emergency admission.

The models explored whether there was an association between each outcome of interest and the fiscal year of admission, adjusting for potentially confounding case mix variables and using robust standard errors (rSE) to appropriately account for multiple admissions per patient.^23^ The adjusted odds ratios for the exposure variable (fiscal year of admission) reflect the change in odds from one year to the next over the course of the observation period, assuming a linear effect (ie, the average change). Adjusted odds ratios below 1.00 indicate a year‐on‐year reduction in risk for the specified outcome.

#### 2.6.3 Sensitivity analysis focusing on longer stay admissions

The cohort was reduced to include only those patients with a UC‐specific emergency admission lasting four or more days, as previously described.^24^ This focuses on more severe cases, and hence those mostly likely to have acute severe colitis and a greater risk of adverse outcomes. All the statistical models were replicated on this selected cohort.

#### 2.6.4 Analysis of regional variation in outcomes over time

We aggregated admissions for each of the five regions of England, based on Lower Super Output Area of residence for each patient at the time of admission. Case mix adjusted rates (indirectly standardised for age, gender and co‐morbidity) for each region were calculated and we generated heat maps to illustrate regional variation for the first and last three‐year periods.

#### 2.6.5 Analysis of inter‐institutional variation in outcomes over time

After examining national and regional level trends, we investigated whether there had been a reduction in institutional variation for key outcomes across England. We identified a subset of all NHS providers (acute NHS Trusts) that were represented consistently in the dataset over the observation period. We excluded provider organisations that were not present in every data year due to changes in constituent hospital units, identifying 136 provider organisations across England. We aggregated admissions to each NHS Trust for the three 3‐year periods, calculating hospital‐specific crude and case‐mix adjusted rates of in‐hospital mortality and emergency surgery. Variation at the level of individual organisations was further explored using funnel plots,^25^ as previously described.^26^ To establish evidence of system wide improvement in care, we stipulated that the absolute number of outlier organisations should decline consistently over each of the three 3‐year periods. We defined an outlier as any provider with an adjusted rate above the 2 SD upper limit for each metric.

## 3 RESULTS

### 3.1 Demographic and clinical characteristics

Table 1 summarises the cohort characteristics, both at admission and patient level (for individual analysis, cases were defined at the time of first UC‐specific emergency admission). Most cases (87%) were coded with K51.9 (Ulcerative colitis, unspecified) rather than codes indicating disease extent. In the national cohort, there were 44 882 UC‐specific emergency admissions involving 32 067 patients. Over three quarters of cases in each cohort had just one index admission over the entire period, and fewer than 10% had more than two. At national level, the case mix was remarkably similar across all years (Table S1).

**Table 1:** Characteristics of emergency admissions for ulcerative colitis, and individual patients, admitted to hospitals in England over a 9‐year period (fiscal years 2005/06 to 2013/14)

Variables	National cohort		Admissions ≥4 d length of stay		136 NHS Trusts^a^	
	Admissions n = 44 882	Patients n = 32 067	Admissions n = 32 148	Patients n = 24 900	Admissions n = 41 250	Patients n = 29 577
Age, mean (SD)	46 (20)	47 (20)	48 (21)	49 (21)	46 (20)	47 (20)
Age groups, %						
≤29	26.8	24.7	24.6	23.5	26.6	24.6
30‐49	33.3	32.4	31.6	31.1	33.3	32.4
50‐69	23.0	24.3	24.0	24.7	23.2	24.5
70+	17.0	18.6	19.8	20.8	16.9	18.5
Men, %	51.1	51.5	51.9	52.1	51.1	51.4
Co‐morbidity groups (Charlson), %						
0	78.9	78.9	77.2	77.3	78.9	78.9
1 co‐morbidity	17.0	16.9	18.2	18.1	17.0	17.0
2 or more co‐morbidities	4.1	4.1	4.7	4.6	4.1	4.1
Emergency bed days in past year, %						
None	66.2	—	65.3	—	66.2	—
1‐14 nights	24.5	—	24.5	—	24.5	—
15‐28 nights	5.4	—	5.9	—	5.4	—
>28 nights	3.9	—	4.3	—	3.9	—
Quintiles of deprivation index, %						
1 (Most deprived)	20.3	19.5	20.0	19.5	19.8	19.1
2	21.5	20.8	21.6	21.0	21.4	20.7
3	20.8	20.9	20.7	20.7	21.0	21.2
4	19.2	19.5	19.5	19.9	19.3	19.6
5 (Least deprived)	17.3	18.1	17.4	17.9	17.6	18.4

a Includes emergency admissions to 136 providers (NHS Trusts) represented in all fiscal years, selected for analysis of inter‐institutional variation.

**Figure 1:**
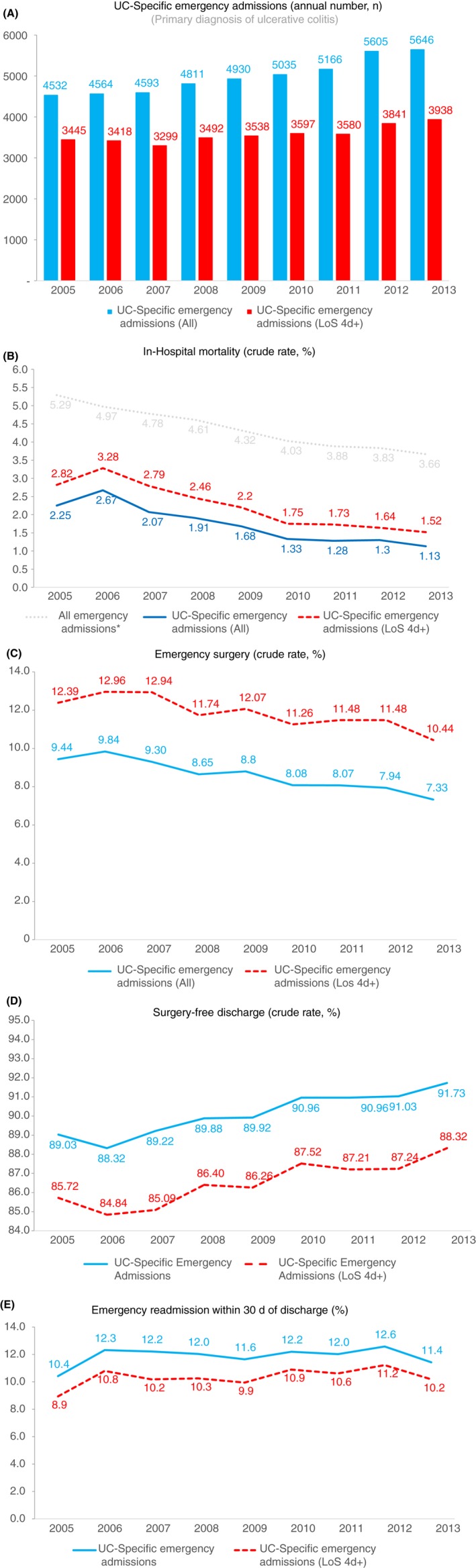
Annual number of emergency admissions with a primary diagnosis of ulcerative colitis to English hospitals (A) and their crude outcomes (B‐E) for financial years 2005‐2013. Data are presented for base case cohort (all admissions, in blue) and the cohort used for sensitivity analysis (admissions with a length of stay of four or more days; LoS 4d+, in red). *Data for crude all‐cause mortality rate derived from Aragón et al^20^

The annual number of admissions increased over time (Figure 1A). The mean (SD) length of stay (LoS) was 10.0 (13.5) days, with a total of 447 696 emergency bed days. There was a gradual decline in LoS from 11.8 (15.8) days in 2005 to 8.7 (11.2) days in 2013. LoS reduced from 10 (13) to 7 (10) days for admissions without surgery and from 30 (24) to 24 (20) days for admissions with emergency surgery. Compared across 3‐year periods, the reduction in mean LoS was significant for non‐surgical admissions (9.3, 8.5 and 7.6 days; *P* < 0.001, ANOVA) and for those with surgery (29.5, 25.9 and 25.3 days; *P* < 0.001, ANOVA).

### 3.2 Crude outcomes and time trends at national level

#### 3.2.1 In‐hospital deaths

About 764 deaths occurred during a UC‐specific emergency admission, giving a crude in‐hospital mortality rate of 1.7% for 2005‐2013 (17 deaths per 1000 admissions). There were 546 deaths during admissions without surgery (n = 41 045 admissions; 14 deaths per 1000 admissions) and 200 in‐hospital deaths after emergency surgery (n = 3837 admissions; 52 deaths per 1000 admissions). Compared with live discharges, admissions with deaths had significantly higher age (mean: 76.8 vs 45.5 years, *P* < 0.0001) and levels of co‐morbidity (Charlson 2+: 22.5% vs 3.8%, *P* < 0.001), but there was no difference in sex (% male: 48.3 vs 51.2, *P* = 0.111) or deprivation status.

Crude in‐hospital death rate declined by half for all UC‐specific emergency admissions, from 2.3% to 1.1% (Figure 1B). As expected, mortality for the sub‐group of admissions lasting four or more days was higher but showed a similar downward trend (2.8%‐1.5%). To compare UC‐specific admissions with overall national trends, Figure 1B also shows annual in‐hospital death rates reported for all‐cause emergency admissions, as reported by Aragón et al*.* 20 The overall decline in crude death rate was less than a third for all‐cause admissions.

#### 3.2.2 Emergency surgery

There were 3837 first major emergency surgeries. Of the 44 882 UC‐specific emergency admissions across 9 years, 43 604 occurred in patients who had not undergone surgery during any previous admission. Hence, the overall crude rate of emergency (first) surgery was 8.8% for 2005‐2013. From year to year, the crude rate of surgery decreased from 9.4% to 7.3% for UC‐specific admissions overall, and from 12.4% to 10.4% for longer stay admissions (Figure 1C).

Primary procedure codes were predominantly for sub‐total‐, total‐ or panproctocolectomy (89%), with the remainder compatible with other major colonic resections and/or stoma, but none for “elective”‐type operations (eg, ileo‐anal pouch), consistent with the unplanned nature of the procedures. The annual proportion of operations with codes indicating a laparoscopic approach increased from 2.1% to 34.5% over the 9‐year period. The trend was significant over consecutive 3‐year periods (3.5% vs 17.9% vs 28.4%; *P* < 0.001, Chi‐Square 3x2 Table). There was also a reduction in the mean time between admission and surgery, from 12.2 (12.1) days in 2005 to 9.3 (6.7) days in 2013—corresponding figures for the 3‐year periods were 11.9 (10.2), 10.6 (8.5) and 10.1 (13.6) days, respectively (*P* < 0.001, ANOVA).

#### 3.2.3 Colectomy‐free discharge

Overall, the crude annual rate of live discharges without colectomy increased from 89% to 92% over the period for all UC‐specific admissions (Figure 1D). The higher mortality and surgical rates among admissions lasting four or more days translate into lower rates of colectomy‐free discharge but the trend of improvement was similar.

#### 3.2.4 Emergency readmission within 30‐days of discharge

There were 5311 unplanned readmissions to hospital within 30 days of discharge following a UC‐specific emergency admission, giving a crude readmission rate of 12.2%. Of the 5311 readmissions, 4089 followed a colectomy‐free discharge and 1222 occurred after an index admission when surgery had been performed. The top 20 primary diagnoses recorded for readmissions are summarised in Table S2, categorized according to whether surgery occurred or not during index admission. As expected, the commonest readmission codes were for ulcerative colitis (over half) or gastrointestinal symptoms. Infections and VTE appeared in the top 20 causes for rehospitalisation following non‐surgical discharges, whereas the list of post‐colectomy readmissions contained several post‐operative complications. Over the years, the crude rate of all‐cause emergency readmission fluctuated but did not decline consistently, both for all admissions and for the sensitivity analysis cohort (Figure 1E).

#### 3.2.5 In‐hospital deaths and emergency surgery during 30‐day readmissions

About 171 in‐hospital deaths occurred during an emergency readmission (3.2% of readmissions had a fatal outcome). Adding this outcome to deaths during index admissions, there were 935 in‐hospital deaths—hence, 2.0% of the original 44 882 emergency admissions died either during their index admission or during an early re‐hospitalization.

Of the original colectomy‐free discharges for UC, there were 517 emergency readmissions with first major surgery performed during the further hospital stay. Hence, 13 per 1000 colectomy‐free discharges were followed by readmission within 30‐days with a requirement for emergency first surgery.

### 3.3 Associations between year of admission and outcomes at national level

**Figure 2:**
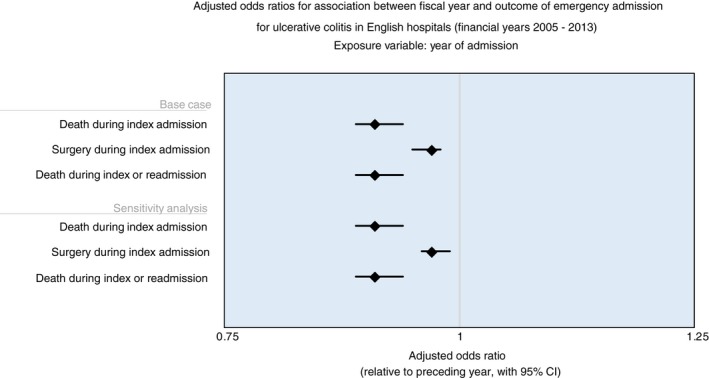
Adjusted odds ratios for association between fiscal year and outcome of emergency admission for ulcerative colitis in English hospitals, 2005‐2013. The figures show case mix adjusted odds ratios (with 95% CIs) for any given year relative to the preceding year. See Tables 2, 3 and 5 for complete model outputs. Sensitivity analysis focuses on patients with length of stay of four or more days (LoS4d+)

The primary focus of our analysis was to determine whether risk‐adjusted odds for key outcomes were associated with fiscal year of admission. Selected model outputs are summarised in Tables 2, 3, 4, 5 and Figure 2, including base‐case and sensitivity analyses.

**Table 2:** Variables associated with in‐hospital mortality following emergency admission to English hospitals for ulcerative colitis (financial years 2005/06 to 2013/14)

Variable	Base case model^a^			Sensitivity analysis^b^		
	Model coefficient (robust SE)	OR	95% CI	Model coefficient (robust SE)	OR	95% CI
Age	0.10 (0.004)	1.11	(1.10, 1.11)	0.10 (0.004)	1.10	(1.09, 1.11)
Year of admission	−0.09 (0.02)	0.91	(0.89, 0.94)	−0.09 (0.02)	0.91	(0.89, 0.94)
Colectomy	1.97 (0.10)	7.20	(5.97, 8.69)	1.81 (0.10)	6.09	(5.02, 7.37)
Emergency bed days in past year						
0 nights (reference)	—	—	—	—	—	—
1‐14 nights	0.05 (0.10)	1.05	(0.87, 1.28)	0.01 (0.10)	1.01	(0.83, 1.23)
15‐28 nights	0.33 (0.13)	1.40	(1.08, 1.80)	0.31 (0.13)	1.36	(1.05, 1.77)
>28 nights	0.70 (0.12)	2.00	(1.57, 2.56)	0.56 (0.13)	1.75	(1.35, 2.26)
Comorbidities						
None (reference)	—	—	—	—	—	—
1	0.62 (0.09)	1.86	(1.55, 2.21)	0.59 (0.09)	1.81	(1.51, 2.18)
2	1.09 (0.12)	2.97	(2.36, 3.73)	1.09 (0.12)	2.99	(2.36, 3.78)

Note

Multivariable models with stepwise selection of variables. Only significant variables included, *P* < 0.001 throughout. Sex and deprivation status were not independently associated with outcome.

Abbreviations: 95% CI, 95% confidence intervals; OR, adjusted odds ratio.

a 764 events from 32 067 patients; 44 882 admissions.

b 713 events from 24 900 patients; 32 148 admissions with length of stay greater than 3 d.

**Table 3:** Variables associated with first major surgery during emergency admission to English hospitals for ulcerative colitis (financial years 2005/06 to 2013/14)

Variable	Base case model^a^			Sensitivity analysis^b^		
	Model coefficient (robust SE)	OR	95% CI	Model coefficient (robust SE)	OR	95% CI
Age	n/a	n/a	n/a	−0.005 (0.001)	0.995	(0.993, 0.997)
Female	−0.30 (0.03)	0.74	(0.69, 0.80)	−0.25 (0.04)	0.78	(0.73, 0.83)
Year of admission	−0.04 (0.01)	0.97	(0.95, 0.98)	−0.03 (0.01)	0.97	(0.96, 0.99)
Emergency bed days in past year						
0 nights (reference)	—	—	—	—	—	—
1‐14 nights	0.28 (0.04)	1.33	(1.23, 1.43)	0.28 (0.04)	1.32	(1.22, 1.43)
15‐28 nights	0.65 (0.07)	1.91	(1.67, 2.18)	0.60 (0.07)	1.82	(1.59, 2.08)
>28 nights	0.08 (0.10)	1.08	(0.88, 1.32)	−0.0002 (0.10)	1.00	(0.82, 1.22)
Quintile of deprivation						
5 Most deprived (reference)	—	—	—	—	—	—
4	−0.01 (0.05)	0.99	(0.89, 1.09)	−0.03 (0.05)	0.97	(0.87, 1.08)
3	−0.06 (0.05)	0.94	(0.85, 1.04)	−0.07 (0.05)	0.94	(0.84, 1.04)
2	−0.17 (0.05)	0.84	(0.76, 0.94)	−0.18 (0.05)	0.83	(0.75, 0.93)
1 (Least deprived)	−0.34 (0.06)	0.71	(0.63, 0.79)	−0.34 (0.06)	0.71	(0.63, 0.80)
Comorbidities						
None (reference)	n/a	n/a	n/a	—	—	—
1	n/a	n/a	n/a	−0.01 (0.05)	0.99*	(0.90, 1.08)
2	n/a	n/a	n/a	−0.26 (0.10)	0.77*	(0.63, 0.93)

Note

Multivariable models with stepwise selection of variables. Only significant variables included, *P* < 0.001 throughout with exception of: *P = 0.030.

Abbreviations: 95% CI, 95% confidence intervals; OR, adjusted odds ratio.

a 3837 events from 31 535 patients; 43 604 UC specific emergency admissions (excluding prior surgery). Age and comorbidities were not independently associated with outcome.

b 3802 events from 24 527 patients; 31 390 UC specific emergency admissions (excluding prior surgery).

**Table 4:** Variables associated with emergency readmission within 30 d of discharge following an unplanned admission for ulcerative colitis to English hospitals (financial years 2005/06 to 2013/14)

Variable	Base case model^a^			Sensitivity analysis^b^		
	Model coefficient (robust SE)	OR	95% CI	Model coefficient (robust SE)	OR	95% CI
Age	−0.003 (0.001)	0.997	(0.996, 0.999)	n/s	n/s	n/s
Female	n/s	n/s	n/s	−0.10 (0.04)	0.91**	(0.84, 0.98)
Year of admission	n/s	n/s	n/s	0.02 (0.007)	1.02***	(1.00, 1.03)
Colectomy during index admission	−0.13 (0.06)	0.88*	(0.79, 0.98)	n/s	n/s	n/s
Emergency bed days in past year						
0 nights (reference)	—	—	—	—	—	—
1‐14 nights	0.38 (0.03)	1.46	(1.36, 1.56)	0.36 (0.04)	1.43	(1.32, 1.55)
15‐28 nights	0.53 (0.06)	1.70	(1.50, 1.93)	0.44 (0.07)	1.56	(1.35, 1.80)
>28 nights	0.97 (0.10)	2.65	(2.19, 3.20)	0.81 (0.08)	2.25	(1.92, 2.64)
Comorbidities						
None (reference)	—	—	—	n/s	n/s	n/s
1	0.08 (0.05)	1.09	(0.99, 1.20)	n/s	n/s	n/s
2	0.27 (0.08)	1.31	(1.12, 1.53)	n/s	n/s	n/s

Note

Multivariable models with stepwise selection of variables. Only significant variables included, *P* < 0.005 throughout with exception of: *P = 0.022, **P = 0.013 and ***P = 0.028.

Abbreviations: 95% CI, 95% confidence intervals; OR, adjusted odds ratio.

a 5311 events from 43 681 live discharges for UC specific emergency admissions. Female gender was not independently associated with outcome.

b 3315 events from 31 143 live discharges for UC specific emergency admissions. Colectomy and co‐morbidities were not independently associated with outcome.

**Table 5:** Variables associated with death during index admission or 30‐day readmission following an unplanned admission for ulcerative colitis to English hospitals (financial years 2005/06 to 2013/14)

Variable	Base case model^a^			Sensitivity analysis^b^		
	Model coefficient (robust SE)	OR	95% CI	Model coefficient (robust SE)	OR	95% CI
Age	0.10 (0.003)	1.10	(1.09, 1.11)	0.09 (0.003)	1.10	(1.09, 1.10)
Year of admission	−0.09 (0.01)	0.91	(0.89, 0.94)	−0.09 (0.02)	0.91	(0.89, 0.94)
Colectomy	1.69 (0.09)	5.42	(4.54, 6.47)	1.54 (0.09)	4.68	(3.90, 5.61)
Emergency bed days in past year						
0 nights (reference)	—	—	—	—	—	—
1‐14 nights	0.15 (0.09)	1.16	(0.98, 1.37)	0.07 (0.09)	1.08	(0.90, 1.29)
15‐28 nights	0.35 (0.12)	1.42	(1.12, 1.80)	0.33 (0.12)	1.40	(1.10, 1.77)
>28 nights	0.68 (0.12)	1.97	(1.57, 2.48)	0.55 (0.12)	1.73	(1.37, 2.20)
Comorbidities						
None (reference)	—	—	—	—	—	—
1	0.58 (0.08)	1.79	(1.52, 2.11)	0.56 (0.09)	1.76	(1.48, 2.08)
2	1.06 (0.11)	2.87	(2.33, 3.54)	1.07 (0.11)	2.92	(2.35, 3.62)

Note

Multivariable models with stepwise selection of variables. Only significant variables included, *P* < 0.001 throughout.

Abbreviations: 95% CI, 95% confidence intervals; OR, adjusted odds ratio.

a 935 events from 32 067 patients; 44 882 UC specific emergency admissions.

b 856 events from 24 900 patients; 32 148 UC specific emergency admissions.

#### 3.3.1 In‐hospital death during index UC‐specific admission

After adjusting for case mix factors, we confirmed that year of admission was associated with a significant reduction in odds for in‐hospital death (adjusted OR 0.91, Table 2)—hence, assuming a linear effect, the odds of dying in hospital for any given year was 9% lower than the preceding year. Restricting the analysis to UC‐specific admissions lasting 4 or more days in sensitivity analysis resulted in identical findings (Table 2; Figure 2).

We constructed two further models of in‐hospital mortality for index UC‐specific admissions. Firstly, we focused on UC‐specific admissions where surgery was not performed (546 death events), confirming an independent association between year of admission and death (model coefficient: −0.07 (rSE: 0.02); adjusted OR: 0.93; 95% CI: 0.90, 0.97; *P* < 0.001). Secondly, we examined UC‐admissions where first major surgery was undertaken (200 death events from 3837 admissions with surgery), which also showed a reduced risk over successive years (model coefficient: −0.17 (rSE: 0.03); adjusted OR: 0.84; 95% CI: 0.79, 0.90; *P* < 0.001). Hence, declining risk of in‐hospital mortality from year to year was confirmed both for cases managed medically and for those requiring emergency surgery.

#### 3.3.2 Emergency surgery during index UC‐specific admission

Both the base case and sensitivity analysis confirmed a reduction in odds of first major surgery occurring during emergency admission for colitis (adjusted OR 0.97, Table 3; Figure 2)—hence, 3% lower odds for any year compared to preceding year.

#### 3.3.3 Colectomy‐free discharge

Combining death and/or surgery during index admission as a composite outcome confirmed a year‐to‐year decline in risk, both in base case model (model coefficient: −0.04 (rSE: 0.01); adjusted OR: 0.96; 95% CI: 0.95, 0.97; *P* < 0.001) and the cohort of longer stay admissions (model coefficient: −0.03 (rSE: 0.01); adjusted OR: 0.97; 95% CI: 0.96, 0.98; *P* < 0.001). The reciprocal of this composite outcome is colectomy‐free discharge. Hence, the odds of being discharged alive without undergoing emergency surgery for any given year was 3%‐4% higher than the last.

#### 3.3.4 All‐cause emergency readmission within 30 days of discharge

After adjusting for case mix variables, there were no significant association between year of admission and odds for all‐cause readmission in the base case model (Table 4). Hence, among patients discharged alive there was no “year effect.” For the sub‐group of patients with longer stays, year of admission was actually associated with slight increase in odds for readmission (adjusted OR 1.02). Separate models focusing either on non‐surgical or surgical index admissions found no evidence for declining risk of readmission with year of treatment.

#### 3.3.5 Composite outcomes during index and 30‐day readmissions

Models incorporating any additional deaths during unplanned readmission confirmed a lowering of odds of mortality associated with year of admission, both in the base case (adjusted OR 0.91) and in the sensitivity analysis of longer stay admissions (adjusted OR 0.91), as shown in Table 5 and Figure 2. Similarly, a reduction in odds for emergency surgery was confirmed in models incorporating first surgery during index or unplanned readmission in the base case (model coefficient: −0.03 (rSE: 0.01); adjusted OR: 0.97; 95% CI: 0.95, 0.98; *P* < 0.001) and sensitivity analysis (model coefficient: −0.03 (rSE: 0.01); adjusted OR: 0.97; 95% CI: 0.96, 0.90; *P* < 0.001). Although we found no reduction in the odds of being readmitted, the risk of adverse outcomes (death and surgery) was reduced.

### 3.4 Associations between case mix variables and primary outcomes

The case mix factors associated with mortality outcomes (Tables 2 and 5) were predictable, with odds of death increasing with age and measures of co‐morbidity (Charlson co‐morbidities and total emergency bed days in preceding year). The sevenfold increase in risk associated with colectomy is expected, as the requirement for surgery identifies those patients with severe, refractory disease or complications. In agreement with previous studies, we observed that the adjusted odds for surgery was significantly lower for female UC patients 27 and was higher among more affluent patients^21^ (Table 3). However, these associations for surgery did not translate into inequality in mortality outcomes. As expected, patients with high levels of co‐morbidity had a substantially greater risk of all‐cause emergency readmission (Table 4). Overall, post‐surgical patients who survived index admission had a lower odds for unplanned readmission than patients discharged without surgery.

### 3.5 Temporal trends for specific causes of 30‐day emergency readmission

**Figure 3:**
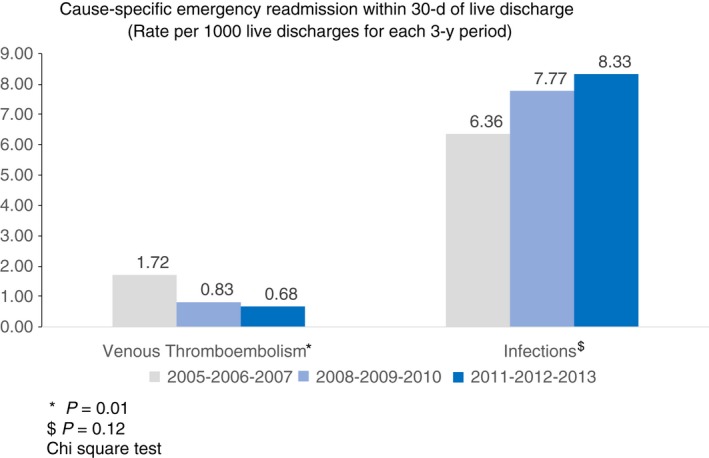
Rates of cause‐specific emergency readmissions within 30‐days of live discharge for venous thromboembolism (VTE) and infections, comparing three consecutive 3‐year periods between 2005 and 2013. There was a significant reduction in rates of readmission for VTE over time

Having found no overall reduction in risk of all‐cause emergency readmission, we examined whether there had been changes in readmission rates for two specific causes (VTE and infections) by comparing rates for successive 3‐year periods. The readmission rate for VTE declined significantly from 1.72 to 0.68 per 1000 live discharges (*P* = 0.01, chi‐squared test) whereas that for infection increased from 6.36 to 8.33 per 1000 live discharges but did not reach statistical significance (*P* = 0.12), Figure 3.

### 3.6 Temporal trends in outcome across the five regions of England

Adjusted rates of in‐hospital death and first major surgery for residents of each of the five regions of England are shown in Figure S1. This shows that rates declined in all regions between the baseline and final 3‐year periods.

### 3.7 Temporal trends in institutional‐level variation

**Figure 4:**
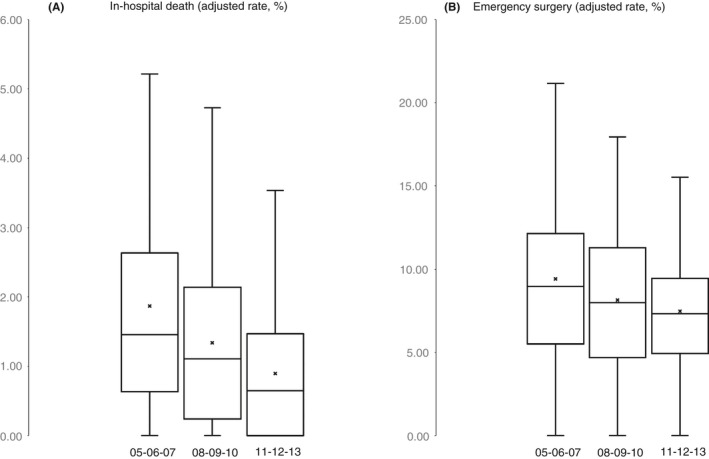
Summary statistics for distribution of adjusted institutional‐level rates of (A) in‐hospital death and (B) emergency surgery for non‐elective admissions for ulcerative colitis across 136 English hospitals (NHS Trusts) over three consecutive 3‐year periods between 2005/2006 to 2013/2014. Box‐whisker plot shows range (error bars), interquartile range (IQR) (box), median (central bar) and mean (x) values. For both metrics, there was a significant reduction in mean and median values (*P* < 0.001 for all comparisons) over the three periods, and a narrowing of the absolute range and IQR. This suggests both improved overall nationwide performance and a reduction in inter‐institutional variation

There were 136 organisations (NHS Trusts) eligible for inclusion in the analysis of time trends in inter‐institutional variation, which included 41 250 admissions for 29 577 patients (92% of the national patient cohort). Patient characteristics were almost identical to the main national cohort (Table 1). The geographical location of organisations and place of residence of the admitted cases confirms nationwide coverage (Figure S2). As expected, the mean overall adjusted rates of in‐hospital mortality and emergency surgery across the 136 Trusts declined progressively over the consecutive 3‐year periods (Figure 4). However, this analysis also illustrates that there was a reduction in the absolute range of values, inter‐quartile ranges and standard deviations—suggesting a narrowing of the degree of inter‐institutional variation.

**Figure 5:**
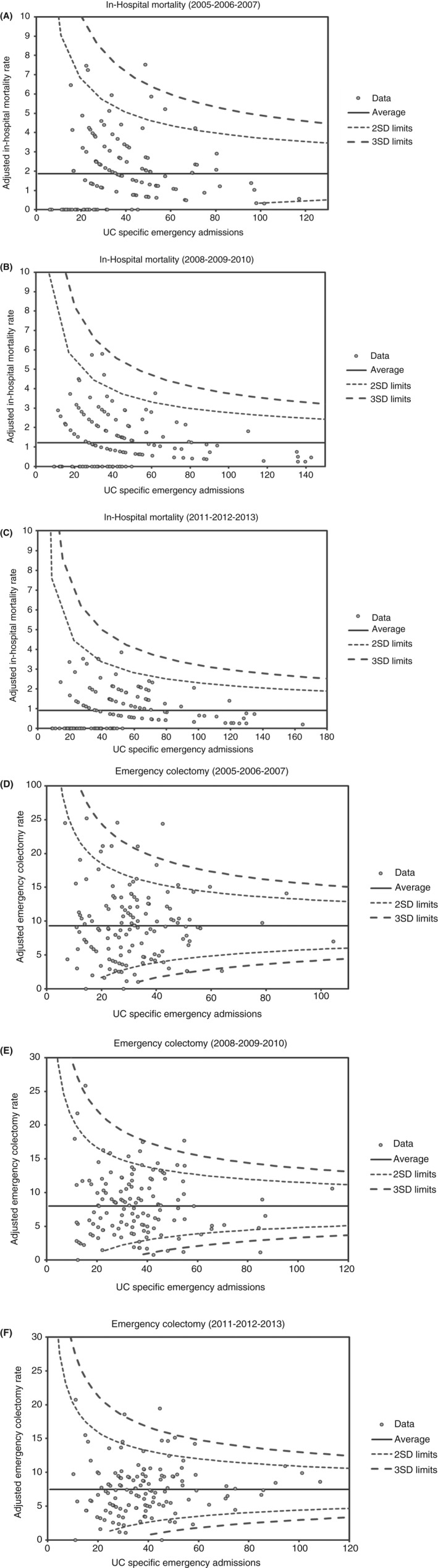
Funnel plots for adjusted institutional‐level rates of in‐hospital death (A‐C) and emergency surgery (D‐F) vs number of UC‐specific admissions across 136 English hospitals (NHS Trusts) for three consecutive 3‐year periods between 2005/2006 to 2013/2014. For both metrics, there was a step‐wise reduction in the number of “outlier” organisations over time. SD, standard deviation. Outliers were defined as organisations with adjusted rates above the 2 SD control limit

We further explored variation between individual providers by constructing funnel plots for case mix‐adjusted in‐hospital mortality (Figure 5A‐C) and compared them across the three time periods. There was stepwise reduction in the number of “outliers” for in‐hospital mortality, from seven (5.1%), through four (2.9%), to two (1.4%) organisations, respectively. Corresponding funnel plots for rates of emergency surgery during index admission are shown in Figure 5D‐F. Again, the number of organisations with adjusted rates of surgery above the 2 SD control limit declined, from 12 (8.8%), through 11 (8.1%) to 8 (5.9%) organisations, respectively. This reduction in dispersion and counts of outliers over time provides further evidence for a nationwide reduction in the prevalence of unexplained variation between providers. Taken together, these data confirm health service wide improvements in care outcome over the observation period.

## 4 DISCUSSION

We analysed administrative data for 44 882 emergency admissions for UC to English hospitals between 2005 and 2013, exploring national, regional and institutional level trends for key outcomes. Over this period, a halving of crude in‐hospital mortality had been reported among cases submitted to the UK IBD audit (1.7%‐0.75%). Although we found that crude death rates for England were somewhat higher than in the sample captured by the audit, we confirmed a reduction by half (2.3%‐1.1%). Moreover, after adjustment for case mix, there was a 9% year‐on‐year reduction in odds of in‐hospital death. Multiple models and sensitivity analyses confirmed this statistically significant improvement in risk‐adjusted mortality outcomes, which held true for cases managed medically and those requiring emergency surgery. Improvements occurred across all regions of England. Although institutional‐level comparisons of death rates for low mortality conditions require caution,^26^ the observed reduction in statistical outliers around a decreasing national average provides strong evidence for a decline in unwarranted variation between centres.

The IBD Audit did not establish whether rates of emergency surgery had changed significantly. However, we observed a fall in crude rate from 9.4% to 7.3% and our models confirmed a 3% year‐on‐year reduction in adjusted odds of unplanned first surgical intervention for colitis. We believe this change is likely to reflect a reduction in “avoidable” surgery rather than failure to offer timely colectomy in life‐threatening situations. The former interpretation is supported by the downward trends observed in risk of in‐hospital mortality for both medical and surgical cases, including the models incorporating deaths and surgery during 30‐day readmissions. Furthermore, the observed reduction by 1‐2 days in mean time to surgery suggests more timely urgent operations when required. Regional and institutional analyses suggest that reductions in avoidable emergency surgery occurred across the country. These trends for unplanned surgery in England are very similar to those reported from the USA.^28^ Analysis of the National Emergency Department Sample (NEDS) revealed that crude rates of surgery declined from 13.4% to 7.8% between 2006 and 2014 for cases of UC admitted via the emergency department. Murthy et al reported lower crude rates of colectomy (below 6%) among 4278 UC patients hospitalised in Ontario between 2002 and 2008.^11^

The large increase we observed in the percentage of operations performed laparoscopically is consistent with the findings of the UK IBD Audit, which noted a sevenfold increase in units reporting availability of minimally invasive non‐elective surgery between 2006 and 2010.^4^, ^6^

Early readmission is regarded as a key marker of care quality internationally.^29^ Despite finding reductions in mortality and surgery risk across both index and readmissions, the crude rate of re‐hospitalization for UC remained stubbornly stable at around 12%. Models showed no reduction in odds for readmission over time, irrespective of whether surgery was needed or not. This is disappointing and suggests a key area for future quality improvement. Comparable published data for longitudinal trends in rates of readmission from other countries are sparse. However, a 30‐day readmission rate of 10.6% was reported from the United States of America in 2013, based on analysis of 26 094 admissions coded with UC as the primary diagnosis in the National Readmission Database (NRD).^30^ As in our study, more than half the readmissions in the USA were coded as primarily related to UC. We identified that post‐surgical patients had a lower odds for readmission compared to unoperated cases, but this trend was not seen in the American study.^30^ Our further analysis of re‐hospitalisation for VTE and infections suggests that the reasons for readmission have evolved over time—with a reduction in readmission rate for VTE but a trend towards an increased rate for infections.

Future quality improvement activities need to include standards for aftercare in the immediate post‐discharge period to reduce avoidable readmissions. In a study of repeat hospitalizations in veterans with IBD, lack of an elective follow‐up visit after discharge was an independent risk factor for 90‐day readmission.^31^ The UK IBD Audit did not include any process measures relating to early post‐discharge review.

Analysis of administrative data has inevitable limitations, including a lack of information about physiological status or disease severity in discharge coding. However, careful analysis has been shown to rival clinical databases for predicting in‐hospital mortality in some other conditions.^32^ We went beyond the use of standard case mix variables (age, gender, co‐morbidity) by constructing an additional proxy measure of each patient's overall comorbid status—summing emergency bed days in the whole year before each admission. We cannot exclude residual confounding due to unmeasured case mix factors, but this seems unlikely to have systematically biased the results in favour of our main findings for time trends. Furthermore, we examined multiple models of individual and composite outcomes and replicated all key findings in sensitivity analyses focused on longer stay cases. The analyses restricted to index admissions lasting at least 4 days is crucial. This focuses on admissions that are likely to be for severe colitis,^24^ as confirmed by the higher rates of mortality and surgery observed. This mitigates the risk of confounding due to a selective rise in admissions of “milder” cases with short stays—a potential criticism had we only undertaken the base case analysis. Lack of drug coding in HES precluded analysis of inpatient therapies or discharge medications. Although there is significant emphasis on volume‐outcome relationships in the surgical literature, we did not try to examine whether high volume surgical centres had “better” post‐operative outcomes. High volumes of emergency surgery at institutional level may reflect sub‐optimal medical management, which may impact adversely on downstream surgical outcomes. Furthermore, it is difficult to disaggregate data for multi‐site providers (large NHS Trusts) where surgical caseload may vary across constituent hospitals and teams. This is an important question for future research.

Increasing digitalisation of healthcare and data linkages to disease registries offers potential for systematic, prospective collection of richer standardised datasets as part of routine care delivery.^33^ International efforts to define common standardised datasets for IBD are growing.^13^ However, for the foreseeable future, metrics derived from administrative data are likely to remain a key source of nationally representative data for studying temporal trends and benchmarking.

We focused on metrics of emergency hospital care and traditional 30‐day post‐discharge period, rather than longer term events. Colectomy‐free discharge after emergency admission for ulcerative colitis is a key therapeutic goal of modern medical treatment and the UK quality improvement programme was focused largely on inpatient care.^4^, ^5^, ^6^, ^7^ Our aim was to generate metrics suited to analysing institutional level performance based on pooled data over consecutive years. Hence, our basic denominators were counts of admissions and our numerators were events related to those admissions. Our models applied robust standard errors to account for readmissions within the same patient,^23^ exploiting the full potential of the dataset. We avoided population‐based metrics (expressing outcomes per‐capita of the general population), since these are unsuited to comparisons between hospitals (which lack a well‐defined catchment population).

It is not possible to prove a causal link between the UK IBD Audit programme per se and improved outcomes. The factors contributing to these trends are complex and will reflect general improvements in emergency services and patient safety, as well as improvements in UC‐specific care. We explored the potential to compare outcomes for hospitals that did, or did not, participate in the IBD audit. However, very few institutions failed to participate in the programme, and we found case numbers and event rates were too small to make meaningful comparisons. Regardless of the reasons for better outcomes, these trends are good news for patients. Our findings for institutional variation show that it mattered less “where” patients were admitted towards the end of the observation period than at the beginning.

The patient factors associated with outcomes in our models were largely as predicted—such as increasing odds of mortality with age, co‐morbidity and the need for emergency surgery. The lower odds of colectomy among female patients and higher odds among more affluent patients are consistent with the previous reports.^21^, ^27^ A range of factors might explain these associations, such as gender‐ or socioeconomic‐related variation in the seeking of care or in admission threshold (ie, differences in severity of disease), or differences between acceptability or access to surgery in the emergency phase. However, we found no association between gender or socioeconomic status and risk of in‐hospital death for index admissions (nor in models including deaths during readmissions). Interestingly, among UC patients there was no increased risk of readmission with age, deprivation status or gender but, as expected, levels of co‐morbidity and emergency bed days in the last year were strongly associated with re‐hospitalization.

## 5 CONCLUSIONS

This study shows a significant decline in risk of death and unplanned first surgery for UC patients admitted as emergencies to English hospitals between 2005 and 2013, with a step‐wise reduction in inter‐institutional variation in outcome. Various factors will have contributed to these encouraging trends but the audit programme is likely to have been one driver. With closure of the UK IBD Audit, it remains to be seen whether standards can be maintained or improved in future. There is no room for complacency—over one in 10 patients were readmitted as an emergency within 30 days of discharge, with no evidence for a reducing risk from year to year.

## AUTHORSHIP

*Guarantor of the article*: Keith Bodger.

*Author contributions*: KB, MS and SD wrote the manuscript. KB, MS and SD designed the research with input from other authors. MS and SD analysed the data, with input from RG, CK and PD. All authors contributed to review of the manuscript and approved the final manuscript.
