# Supplementary material for: Nationwide improvement in outcomes of emergency admission for ulcerative colitis in England, 2005‐2013
Source: Aliment Pharmacol Ther. 2019 May 28;50(2):176–92. doi: 10.1111/apt.15315 (PMC6617780; doi:10.1111/apt.15315)
Supplement: Supplementary file 1 [file APT-50-176-s001.docx]

**Supplementary Table 1 (S1)**

**Table S1**

**Supplementary Table 2 (S2)**

**Top twenty primary diagnoses recorded for 30-day emergency readmissions after index admission for ulcerative colitis, categorised according to surgical outcome of the original admission**

| **No Surgery During Index Admission** | | | **Surgery During Index Admission** | | |
| --- | --- | --- | --- | --- | --- |
| **Code** | **Primary Diagnosis** | **%** | **Code** | **Primary Diagnosis** | **%** |
| K519 | Ulcerative colitis, unspecified | 45.1 | K519 | Ulcerative colitis, unspecified | 51.9 |
| K529 | Noninfective gastroenteritis and colitis, unspecified | 2.7 | T814 | Infection following a procedure, not elsewhere classified | 3.4 |
| K512 | Ulcerative (chronic) proctitis | 2.2 | K914 | Colostomy and enterostomy malfunction | 3.0 |
| K513 | Ulcerative (chronic) rectosigmoiditis | 2.0 | R104 | Other and unspecified abdominal pain | 2.8 |
| R104 | Other and unspecified abdominal pain | 1.9 | K518 | Other ulcerative colitis | 2.6 |
| K518 | Other ulcerative colitis | 1.7 | K510 | Ulcerative (chronic) enterocolitis | 2.6 |
| K510 | Ulcerative (chronic) enterocolitis | 1.6 | T813 | Disruption of operation wound, not elsewhere classified | 2.3 |
| K590 | Constipation | 1.2 | K513 | Ulcerative (chronic) rectosigmoiditis | 2.1 |
| A047 | Enterocolitis due to Clostridium difficile | 1.1 | K566 | Other and unspecified intestinal obstruction | 2.0 |
| K625 | Haemorrhage of anus and rectum | 1.0 | K512 | Ulcerative (chronic) proctitis | 1.3 |
| N390 | Urinary tract infection, site not specified | 0.9 | K625 | Haemorrhage of anus and rectum | 1.3 |
| J181 | Lobar pneumonia, unspecified | 0.8 | T818 | Other complications of procedures, not elsewhere classified | 1.2 |
| K515 | Mucosal proctocolitis | 0.8 | K650 | Acute peritonitis | 0.9 |
| I269 | Pulmonary embolism w/o mention of acute cor pulmonale | 0.7 | K913 | Postoperative intestinal obstruction | 0.9 |
| R11X | Nausea and vomiting | 0.7 | N390 | Urinary tract infection, site not specified | 0.8 |
| J189 | Pneumonia, unspecified | 0.7 | R11X | Nausea and vomiting | 0.8 |
| R074 | Chest pain, unspecified | 0.6 | R103 | Pain localized to other parts of lower abdomen | 0.8 |
| D649 | Anaemia, unspecified | 0.6 | T810 | Haemorrhage and haematoma complicating a procedure NEC | 0.7 |
| R51X | Headache | 0.6 | K918 | Other postprocedural disorders of digestive system NEC | 0.7 |
| I802 | Phlebitis/thrombophlebitis, deep vessels lwr extremities | 0.6 | K515 | Mucosal proctocolitis | 0.6 |

(n=4,089 unplanned readmissions within 30 days of colectomy-free discharge) (n=1,222 unplanned readmissions within 30 days of post-colectomy discharge)

**Legends to Supplementary Figures**

**Figure 1S (Supplementary Figure 1)**

**Time trends in adjusted rates of in-hospital mortality (A) and first major surgery (B) following emergency admission for ulcerative colitis for the five regions of England.** Comparison of aggregated data for two three-year periods. Maps on left show fiscal years 2005/6 to 2007/8, those on right show 2011/12 to 2013/14. Rates are indirectly standardised for age, gender and co-morbidity. The regions are: North, Midlands & East, South East, South West and London.

**Figure 2S (Supplementary Figure 2)**

**Geographical distribution of place of residence for 29,577** **patients admitted 41,250 times as an emergency with ulcerative colitis to 136 English hospitals (2005 to 2013).** Blue shaded areas represent area of residence **(**Middle Layer Super Output Areas, MSOAs) of patients admitted as an emergency with ulcerative colitis to one of 136 NHS hospitals (H) between 2005/06 and 2013/14. Each MSOA represents a geographical area with a mean population of 7,200 residents. Boundaries for the five regions of England are also shown.

**Supplementary Figure 1S(A)**

**Supplementary Figure 1S(B)**

**Supplementary Figure 2 (2S)**

**
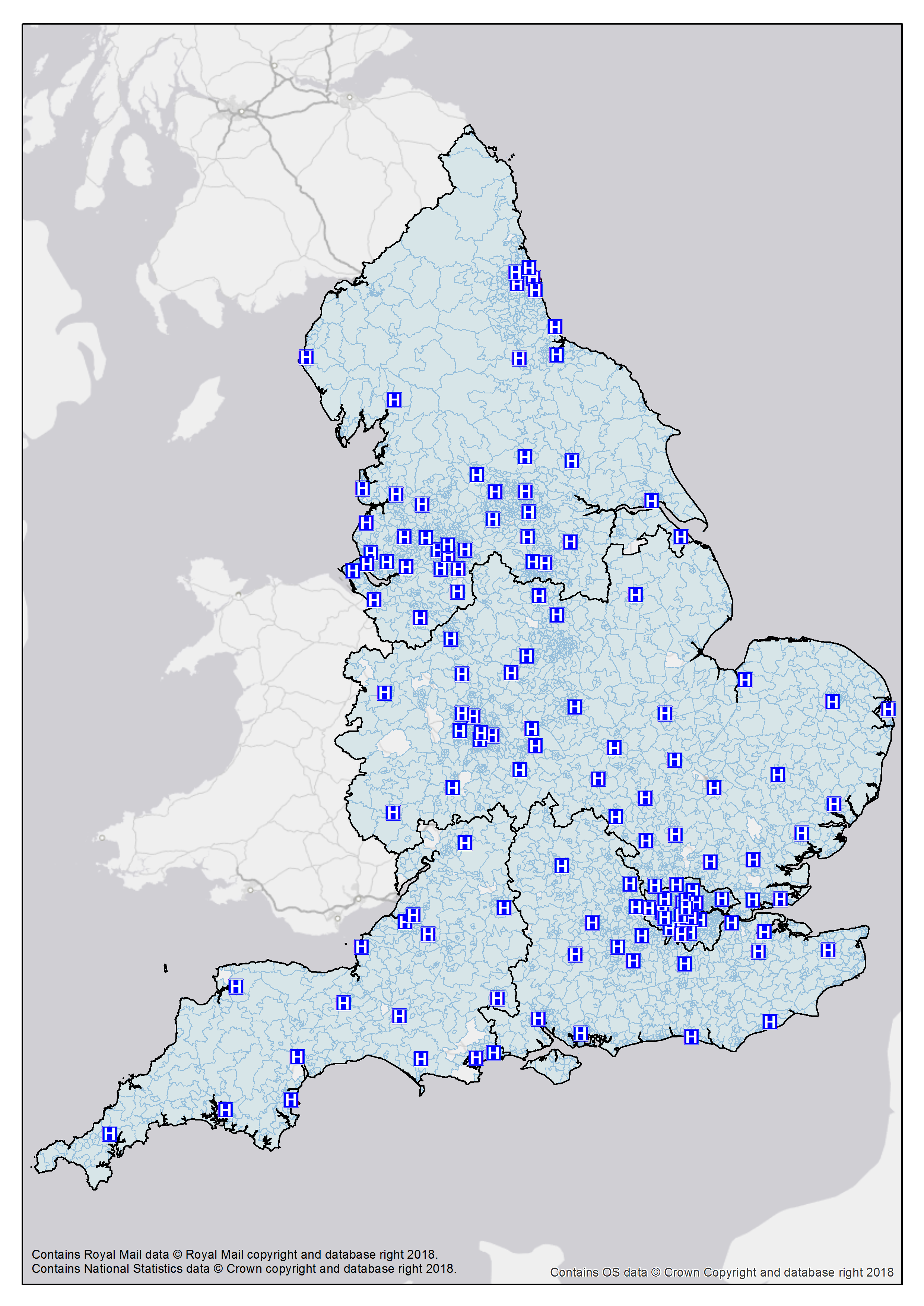
**

Blue shaded areas represent area of residence **(**Middle Layer Super Output Areas, MSOAs) of patients admitted as an emergency with ulcerative colitis to one of 136 NHS hospitals (H) between 2005/06 and 2013/14. Each MSOA represents a geographical area with a mean population of 7,200 residents. Boundaries for the five regions of England are also shown.
